# Accelerated differentiation of human induced pluripotent stem cells to blood–brain barrier endothelial cells

**DOI:** 10.1186/s12987-017-0059-0

**Published:** 2017-04-13

**Authors:** Emma K. Hollmann, Amanda K. Bailey, Archit V. Potharazu, M. Diana Neely, Aaron B. Bowman, Ethan S. Lippmann

**Affiliations:** 1 Department of Chemical and Biomolecular Engineering, Vanderbilt University, 2201 West End Ave, Nashville, TN 37235 USA; 2 Department of Biochemistry, Vanderbilt University, 2201 West End Ave, Nashville, TN 37235 USA; 3 Department of Pediatrics, Vanderbilt University Medical Center, 1211 Medical Center Dr, Nashville, TN 37232 USA; 4 Department of Neurology, Vanderbilt University Medical Center, 1211 Medical Center Dr, Nashville, TN 37232 USA

**Keywords:** Induced pluripotent stem cells (iPSCs), In vitro blood–brain barrier (BBB) model, Brain microvascular endothelial cells (BMECs), Defined differentiation medium

## Abstract

**Background:**

Due to their ability to limitlessly proliferate and specialize into almost any cell type, human induced pluripotent stem cells (iPSCs) offer an unprecedented opportunity to generate human brain microvascular endothelial cells (BMECs), which compose the blood–brain barrier (BBB), for research purposes. Unfortunately, the time, expense, and expertise required to differentiate iPSCs to purified BMECs precludes their widespread use. Here, we report the use of a defined medium that accelerates the differentiation of iPSCs to BMECs while achieving comparable performance to BMECs produced by established methods.

**Methods:**

Induced pluripotent stem cells were seeded at defined densities and differentiated to BMECs using defined medium termed E6. Resultant purified BMEC phenotypes were assessed through trans-endothelial electrical resistance (TEER), fluorescein permeability, and P-glycoprotein and MRP family efflux transporter activity. Expression of endothelial markers and their signature tight junction proteins were confirmed using immunocytochemistry. The influence of co-culture with astrocytes and pericytes on purified BMECs was assessed via TEER measurements. The robustness of the differentiation method was confirmed across independent iPSC lines.

**Results:**

The use of E6 medium, coupled with updated culture methods, reduced the differentiation time of iPSCs to BMECs from thirteen to 8 days. E6-derived BMECs expressed GLUT-1, claudin-5, occludin, PECAM-1, and VE-cadherin and consistently achieved TEER values exceeding 2500 Ω × cm^2^ across multiple iPSC lines, with a maximum TEER value of 4678 ± 49 Ω × cm^2^ and fluorescein permeability below 1.95 × 10^−7^ cm/s. E6-derived BMECs maintained TEER above 1000 Ω × cm^2^ for a minimum of 8 days and showed no statistical difference in efflux transporter activity compared to BMECs differentiated by conventional means. The method was also found to support long-term stability of BMECs harboring biallelic *PARK2* mutations associated with Parkinson’s Disease. Finally, BMECs differentiated using E6 medium responded to inductive cues from astrocytes and pericytes and achieved a maximum TEER value of 6635 ± 315 Ω × cm^2^, which to our knowledge is the highest reported in vitro TEER value to date.

**Conclusions:**

Given the accelerated differentiation, equivalent performance, and reduced cost to produce BMECs, our updated methods should make iPSC-derived in vitro BBB models more accessible for a wide variety of applications.

**Electronic supplementary material:**

The online version of this article (doi:10.1186/s12987-017-0059-0) contains supplementary material, which is available to authorized users.

## Background

The blood–brain barrier (BBB) is composed of brain microvascular endothelial cells (BMECs), which strictly regulate molecular transport between the bloodstream and the brain to ensure proper function of neuronal circuits, synaptic transmission, and other essential processes that depend upon a tightly controlled neurovascular environment [1]. BMECs are characterized by specialized tight junctions [2, 3] that limit paracellular flux of biologics, hydrophilic small molecules, and ions, which results in a characteristically high trans-endothelial electrical resistance (TEER) across the barrier not observed in other capillary beds in the body [4, 5]. For import and export of molecules, BMECs express multiple transporters, such as GLUT-1 [6], and active efflux transporters [6], including P-glycoprotein and multidrug resistance related protein (MRP) family transporters [7], that confer protection. BBB disruption is implicated in many chronic neurodegenerative diseases, such as multiple sclerosis [8], traumatic brain injury [9], ischemic stroke [10–13], and in natural aging of the neurovascular unit [14]. Moreover, the BBB often hinders delivery of therapeutics to diseased tissue when the barrier remains intact [15]. The time, expense, difficulty, and limited throughput of all in vivo research often precludes widespread use of such techniques, necessitating in vitro platforms to investigate certain biological phenomena. Therefore, in vitro BBB models are often employed to study BBB mechanisms, neurovascular cell–cell interactions, and to perform screens for BBB-permeant therapeutics.

In vitro BBB models have most often been constructed using primary BMECs isolated from rat, bovine, and porcine sources [16–21]. Such models extended to BMECs in co-culture with astrocytes, pericytes, and neurons, cell types known to enhance the BBB phenotype and thereby more accurately recapitulate the in vivo neurovascular environment [17, 18]. These models can be used to study BBB development, regulation, and disease, as well as assay potential drug candidates for permeability [19, 20]. However, due to species’ differences in transporter sequences/structures, activities, and expression levels [21], a robust fully-human BBB model is preferred to investigate BBB function and disease in a human context and to perform drug screens that yield the most promising pharmacological compounds for clinical applications.

Human in vitro BBB models have most often utilized either BMECs isolated from primary tissue [22] or immortalized BMEC cell lines [23]. Primary BMECs demonstrate moderate barrier properties, but difficult isolation procedures and low yields hinder their widespread use. Immortalized BMECs provide a readily scalable source of cells for in vitro models, but these cells do not recapitulate the impermeable character of the BBB. Due to their ability to limitlessly proliferate and specialize into any cell type, human induced pluripotent stem cells (iPSCs) offer an unprecedented opportunity to provide human BMECs for research purposes. iPSCs were recently shown to be capable of differentiating to endothelial cells with BBB properties [24]. Though the first-generation differentiation procedure yielded BMECs with passive barrier properties that remained below measurements in animals [4], the addition of retinoic acid (RA) during the differentiation process substantially improved the BBB phenotype, and RA-treated BMECs exhibited TEER reaching near in vivo levels after co-culture with other cell types of the neurovascular unit [25]. Unfortunately, full differentiation from the iPSC state to purified BMECs is a protracted multi-week process and uses complex, expensive maintenance and differentiation medium. The time and cost associated with iPSC culture and this differentiation method are detrimental to widespread use of these BMECs, and an expedited and less costly differentiation process yielding cells of equal performance with significantly less associated expense would alleviate some of these hurdles.

Induced pluripotent stem cell maintenance and differentiation procedures are rapidly evolving towards fully defined compositions [26–29]. In this study, we sought to adopt these procedures in an effort to streamline the BBB differentiation process. We transitioned to recently-described E8 medium for iPSC maintenance [27], as utilized by others for BBB differentiation [30], with no discernible issues. A derivative of E8 medium composition, collectively termed E6 medium (Dulbecco’s modified Eagle’s medium [DMEM]/F-12, ascorbic acid, sodium bicarbonate, selenium, human transferrin, and human insulin) has recently been used for efficient conversion of iPSCs to neuroectoderm [28], and we decided to explore its use in place of unconditioned medium (UM) in the BBB differentiation process. Intriguingly, we discovered that E6 medium, compared to UM, shortens the differentiation timeline without compromising BMEC performance as measured by TEER, sodium fluorescein permeability, and efflux transporter activity [24, 25]. The E6 differentiation procedure was reproducible across several iPSC lines, and upon co-culture with iPSC-derived astrocytes and primary human brain pericytes, BMECs achieved maximum TEER of 6635 ± 315 Ω × cm^2^, which to our knowledge is the highest value ever recorded in any BBB model, and stability of the barrier above 1000 Ω × cm^2^ was observed for 22 days. Overall, we have shortened the differentiation procedure to 8 days with no discernible loss of BBB character. Given that defined medias can be prepared in-house at greatly reduced costs, we suggest that the methods described herein will better enable widespread use of iPSC-derived BMECs.

## Methods

### Media preparation

#### E4 medium

E4 basal medium was prepared in 48 L batches and used to prepare E8 and E6 medium as described below. 48 L of DMEM/F12 with HEPES (Thermo Fisher Scientific, catalog number 11,330,057) was added to a large carboy along with 3.072 g l-Ascorbic acid 2-phosphate sesquimagnesium salt hydrate (Sigma-Aldrich), 931 μL of sodium selenite solution (0.7 mg/mL in PBS; Sigma-Aldrich), and 26.064 g sodium bicarbonate (Sigma-Aldrich). The solution was mixed for 20 min followed by alternating pH and osmolarity tests and adjustments. Osmolarity of the solution was adjusted to 340 ± 5 mOsm/kg using sodium chloride (Fisher Scientific) and tested using a Precision Systems Micro-OSMETTE Model 5004 osmometer. pH of the solution was adjusted to 7.4 ± 0.05 using 5 M sodium hydroxide and measured using a Thermo Scientific Orion Star A211 benchtop pH meter. The solution was mixed for 10 min between each pH and osmolarity adjustment. Final E4 medium was frozen in 500 mL aliquots. Final concentrations in E4 basal medium were 64 mg/L l-Ascorbic acid 2-phosphate sesquimagnesium salt hydrate, 14 μg/L of sodium selenite, and 1743 mg/L sodium bicarbonate (20.75 mM) [27]. Detailed protocols for media preparation are available upon request.

#### E8 medium

E8 medium was prepared by adding 100 μL of human insulin solution (Sigma-Aldrich), 500 μL of 10 mg/mL of human holo-transferrin (R&D Systems), 500 μL of 100 μg/mL human basic fibroblast growth factor (bFGF; Peprotech), and 500 μL of 2 μg/mL TGFβ1 (Peprotech) to 500 mL of E4. The final concentrations are 2.14 mg/L insulin, 100 μg/L bFGF, 2 μg/L TGFβ1, and 10.7 mg/L holo-transferrin [27]. Media was sterile filtered and stored at 4 °C for a maximum of 2 weeks.

#### E6 medium

E6 medium was prepared by adding 100 μL of human insulin solution and 500 μL of 10 mg/mL of human holo-transferrin to 500 mL of E4. The final concentrations are 2.14 mg/L insulin and 10.7 mg/L holo-transferrin [28]. The medium was sterile filtered and stored at 4 °C for a maximum of 2 months.

#### Unconditioned medium (UM)

Unconditioned medium (UM) was prepared as previously described [24]. UM consisted of DMEM/F12 (Thermo Fisher Scientific), 20% Knockout Serum Replacer (Thermo Fisher Scientific), 1× MEM non-essential amino acids (Thermo Fisher Scientific), 1 mM Glutamax (Thermo Fisher Scientific), and 0.1 mM β-mercaptoethanol (Sigma-Aldrich). UM was sterile filtered and stored at 4 °C for a maximum of 2 weeks.

#### Endothelial cell (EC) medium

Endothelial cell (EC) medium consisted of human Endothelial Serum-Free Medium (Thermo Fisher Scientific) supplemented with 1% platelet-poor plasma-derived bovine serum (Fisher Scientific) [31]. EC medium was supplemented with 20 ng/mL bFGF and 10 μM all-trans retinoic acid (Sigma-Aldrich) during the EC phase prior to subculture and during the first 24 h of subculture, then removed to promote induction of the barrier phenotype [25]. EC medium was sterile filtered and stored at 4 °C for a maximum of 2 weeks.

### Maintenance of iPSCs

Cell lines used were IMR90-4 iPSCs [32], CD12 iPSCs [33], CC3 iPSCs [34], and SM14 iPSCs [35, 36]. IMR90-4 and CC3 lines are female. CD12 and SM14 lines are male. All cell lines were thawed directly into E8 medium containing 10 μM Y-27632 (Tocris). 24 h after thaw, cells were changed to E8 medium without Y-27632. E8 medium was changed every day thereafter. All iPSCs were maintained on Matrigel (Corning). Once 70% confluent, iPSCs were passaged by washing cells once with Versene solution (Thermo Fisher Scientific), incubating cells with Versene solution for 5 min at 37 °C, collecting the cells in fresh E8 medium, and distributing the cells at desired ratios to Matrigel-coated plates.

### iPSC differentiation to BMECS

Induced pluripotent stem cell were washed once with DPBS (Thermo Fisher Scientific) and incubated with Accutase (Stem Cell Technologies) for 3 min to yield a single cell suspension. Cells were collected via centrifugation, resuspended in E8, and counted using either a hemocytometer or Countess II (Thermo Fisher Scientific). Trypan blue was not used to measure cell viability when using a hemocytometer but was used when measuring cell density using the Countess II. Cells were seeded at densities ranging from 10,000 to 15,600 cells per square centimeter in E8 containing 10 μM Y-27632 (Day −1). Approximately 24 h after seeding, media was changed to UM or E6 medium (Day 0) to induce differentiation. Media was changed every 24 h. Cells were differentiated in UM for 6 days or in E6 medium for 4 days. Next, cells were treated with EC medium containing 20 ng/mL bFGF and 10 μM retinoic acid for 48 h. Following treatment, EC medium was removed, and cells were washed once with DPBS and incubated with Accutase for 20–25 min [37]. Cells were collected via centrifugation and subsequently purified by selective adhesion onto a collagen-fibronectin matrix. In brief, tissue culture polystyrene plates and Transwell filters (polyethylene terephthalate, 0.4 μm pore size, 1.1 cm^2^ surface area in 12-well format) were coated with a solution of 400 μg/mL collagen IV (Sigma-Aldrich) and 100 μg/mL fibronectin (Sigma-Aldrich) for a minimum of 1 h for plates and a minimum of 4 h up to overnight for Transwell filters. Cells were subcultured onto plates and filters at a ratio of 1 well of a 6-well plate of differentiated cells to 3 wells of a 12-well plate, 6 wells of a 24-well plate, or 3 Transwell filters. To assess barrier properties, TEER was measured 24 h after subculture using an EVOM2 voltohmeter with STX3 chopstick electrodes (World Precision Instruments). Following TEER measurement, cells were changed to EC medium containing no bFGF and no RA to induce barrier phenotype. No further media changes occurred after this point. TEER was measured approximately every 24 h thereafter. Experiments were terminated when average TEER fell below 1000 Ω × cm^2^. All reported TEER values are corrected for the resistance due to an empty Transwell filter.

### Immunocytochemistry

Cells were washed twice with DPBS and fixed for either 20 min in 4% paraformaldehyde (Sigma-Aldrich) or 10 min in 100% ice-cold methanol. Cells were washed 3 times with DPBS and blocked for a minimum of 1 h in PBS or TBS containing 5% donkey serum and 0.3% Triton X-100 (PBS-DT and TBS-DT, respectively). Cells were incubated with primary antibody diluted in PBS or TBS containing 5% donkey serum (PBSD and TBSD, respectively) or in PBS-DT or TBS-DT overnight at 4 °C. Following primary antibody incubation (see Additional file 1: Table S1), cells were rinsed once with PBS or TBS and washed five times with PBS or TBS for a minimum of 5 min per wash. Cells were incubated in secondary antibody (see Additional file 1: Table S2) diluted in the same buffer as primary antibody for a minimum of 1 h. Following secondary antibody incubation, cells were incubated with 4′,6-diamidino-2-pheny-lindoldihydrochloride (DAPI; Thermo Fisher Scientific) for 10 min to label nuclei. Cells were rinsed once and washed five times with PBS/TBS and then visualized using a Zeiss AxioObserver Z1 microscope or a Leica DMi8 microscope. An average of three images were taken for each stain and the entire field was visually assessed to ensure that the presented images are representative of the entire dish.

### Efflux transporter activity assays

#### Substrate accumulation

Induced pluripotent stem cell-derived BMECs were purified into 24-well plates and subjected to EC medium lacking bFGF and RA for 24 h prior to efflux assays. For inhibition experiments, BMECs were incubated with 10 μM PSC833 (Sigma-Aldrich) or 10 μM MK-571 (Sigma-Aldrich) for 1 h at 37 °C. Following this incubation, BMECs were incubated with 10 μM rhodamine 123 (Sigma-Aldrich; 488 nm excitation and 540 nm emission) or 10 μM 2′,7′-dichlorodihydrofluorescein diacetate (H_2_DCFDA, ThermoFisher, 492 nm excitation and 527 nm emission), with or without their respective inhibitors, for 1 h at 37 °C. Cells were washed three times with DPBS and subsequently lysed using DPBS with 5% Triton X-100 to measure dye accumulation in the cells. Fluorescence was measured on a BioTek Synergy H1 multi-mode microplate reader. For each condition, one well of cells was not lysed. These conserved wells were fixed for 10 min in 100% ice-cold methanol and incubated with DAPI for 10 min. Cells were washed three times with DPBS and imaged. 6 images per condition were taken, and nuclei count per culture area was found using CellProfiler analysis software [38]. Fluorescence is reported on a per-cell basis, normalized to control fluorescence from cells treated with fluorescent substrate but no inhibitor.

#### Apical-to-basolateral flux

Induced pluripotent stem cell-derived BMECs were purified onto Transwell filters and subjected to EC medium lacking bFGF and RA for 24 h prior to assays. For inhibition experiments, BMECs were incubated with 10 μM PSC833 or 10 μM MK-571 for 1 h at 37 °C. Inhibitor was only included in the apical chamber. Following this incubation, 10 μM rhodamine 123 or 10 μM H_2_DCFDA was added to the apical chamber, with or without respective inhibitors, for 1 h at 37 °C. 200 μL of media was then removed from the basolateral chamber and fluorescence was measured on a BioTek Synergy H1 multi-mode microplate reader.

### Sodium fluorescein permeability

Induced pluripotent stem cell-derived BMECs were purified onto Transwell filters and subjected to EC medium lacking bFGF and RA for 24 h prior to permeability measurements. Medium was aspirated from the apical and basolateral chambers of each filter and replaced with fresh medium of the same composition to allow for monolayer equilibration. After 1 h, medium from the apical chamber was aspirated and replaced with 0.5 mL of sodium fluorescein (10 μM, Sigma-Aldrich) diluted in fresh medium. Every 30 min, 200 μL of medium was removed from the basolateral chamber and replaced with 200 μL of fresh medium. The same experiment was conducted on an empty filter. After 2 h, fluorescence was measured on a BioTek Synergy H1 multi-mode microplate reader. The rate of accumulation, corrected for media removal and flux across the empty filter, was used to calculate P_e_ values.

### Primary human pericyte culture

Primary human brain vascular pericytes were purchased from ScienCell Research Laboratories. Pericytes were cultured on plates coated with 0.1% gelatin (Sigma-Aldrich) and maintained in DMEM (Corning) supplemented with 10% fetal bovine serum (Thermo Fisher Scientific). Medium was changed every 3 days, and cells were passaged for maintenance when approximately 80% confluent. Pericytes (passage 15) were subcultured onto Matrigel-coated 12-well plates for co-culture experiments when approximately 40% confluent at a ratio of 1 well of a 6-well plate to 3 wells of a 12-well plate. Once co-cultured with iPSC-derived glia, pericytes were maintained in E6 medium with 10 ng/mL CNTF and 10 ng/mL EGF. Pericyte co-culture with BMECs (with or without astrocytes and glial progenitors) was initiated 24 h after BMEC seeding onto Transwell filters. At this time, pericytes were changed to EC medium lacking bFGF and RA. Medium was not changed for the duration of the experiment.

### iPSC differentiation to astrocytes

IMR90-4 iPSCs were washed with 2 mL DPBS and incubated with Accutase for 3 min at 37 °C. Cells were collected via centrifugation, resuspended in E8 medium, and counted using a hemocytometer. Cells were seeded at 2 × 10^5^ cells/cm^2^ onto Matrigel-coated plates in E8 medium containing 10 μM Y-27632. 24 h after seeding, media was changed to E6 medium containing 10 μM SB431542 (Tocris) and 1 μM dorsomorphin dihydrochloride (Tocris). The medium was changed every 24 h for 6 days. On day 6 of differentiation, strips of cells were mechanically picked from the culture using a P200 pipette and transferred to a Matrigel-coated plate with E6 medium containing 10 ng/mL ciliary neurotrophic factor (CNTF; Peprotech), 10 ng/mL epidermal growth factor (EGF; Peprotech), and 10 μM Y-27632. 72 h after picking, media was changed to E6 medium containing 10 ng/mL CNTF and 10 ng/mL EGF but no Y-27632. The medium was changed every 3 days for 30 days. On day 30, cells were incubated with Accutase for 3 min, collected via centrifugation, and seeded at a ratio of 1 well of a 6-well plate to 1 full 6-well plate. Cells were maintained in E6 medium containing 10 ng/mL CNTF and 10 ng/mL EGF with media changes every 48 h. Cells were passaged when approximately 80% confluent. Cells were cultured through a second passage and frozen in liquid nitrogen in E6 medium containing 10% DMSO (Sigma-Aldrich). Frozen iPSC-derived astrocytes were thawed and cultured for two passages before being seeded for co-culture. Astrocytes were seeded for co-culture at a ratio of 1 well of a 6-well plate to 6 wells of a 12-well plate. Cells were maintained in E6 medium containing 10 ng/mL CNTF and 10 ng/mL EGF when in co-culture with pericytes but were switched to EC medium lacking bFGF and RA upon co-culture with BMECs.

### Initiation of co-culture

To initiate co-culture with astrocytes and pericytes, BMECs were subcultured onto Transwell filters as described above. 24 h after subculture, TEER was measured. Transwell filters were then transferred to wells containing astrocytes, pericytes, or astrocytes and pericytes, and medium was changed to EC medium lacking bFGF and RA. BMECs in monoculture served as a control for the experiment. TEER was measured approximately every 24 h. Medium was not changed throughout the duration of the experiment.

### Statistical analysis

Data were expressed as mean ± standard deviation. Two-way ANOVA analysis and Student’s unpaired t test were used to determine statistical significance for efflux transporter activity assays and TEER values.

## Results

### Defined medium shortens required differentiation time to BMECs

**Fig. 1:**
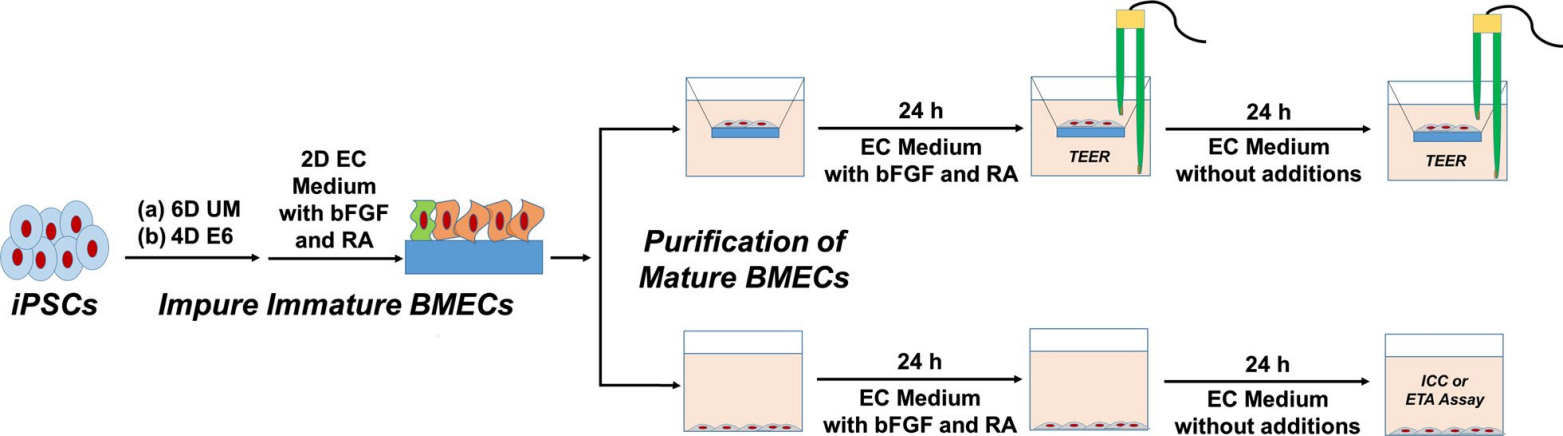
Differentiation of iPSCs to BMECs. iPSCs were seeded at defined densities and differentiated for either 6 days in unconditioned medium (UM) or for 4 days in defined E6 medium. Impure immature BMECs were subsequently treated for 2 days with endothelial cell (EC) medium supplemented with bFGF and retinoic acid (RA). Mature BMECs were purified onto Transwell filters or plates for 24 h in EC medium supplemented with bFGF and RA. 24 h after purification, barrier was induced by treating cells with EC medium lacking bFGF and RA

Previous protocols to differentiate iPSCs to BMECs relied on seeding iPSCs at defined densities, expanding the cells in mTeSR media for 3 days [26], and differentiating the cells for 6 days in UM to immature BMECs [24, 25, 37, 39]. BMECs were expanded in EC medium supplemented with basic fibroblast growth factor (bFGF) and RA and purified onto collagen/fibronectin matrices. Barrier phenotype was then induced by changing to EC medium lacking both bFGF and RA. Peak barrier phenotypes were observed 24 h after this medium change, and it was at this time point that most assays to measure barrier fidelity were conducted. In total, these methods required a minimum of 13 days from time of iPSC seeding to achieving functional, purified BMECs. We sought to improve these established methods in several ways. First, in place of mTeSR, we utilized E8 medium for maintenance [27], as described by others [30]. Next, we removed the 3 day expansion phase prior to differentiation and instead seeded IMR90-4 iPSCs overnight as single cells at densities ranging from 10,000 to 15,600 cells per square centimeter in E8 medium supplemented with Y-27632 before initiating differentiation the following day, a technique that has been utilized in other differentiation protocols [28]. Finally, we explored the use of E6 medium in place of UM during the differentiation process (Fig. 1).

**Fig. 2:**
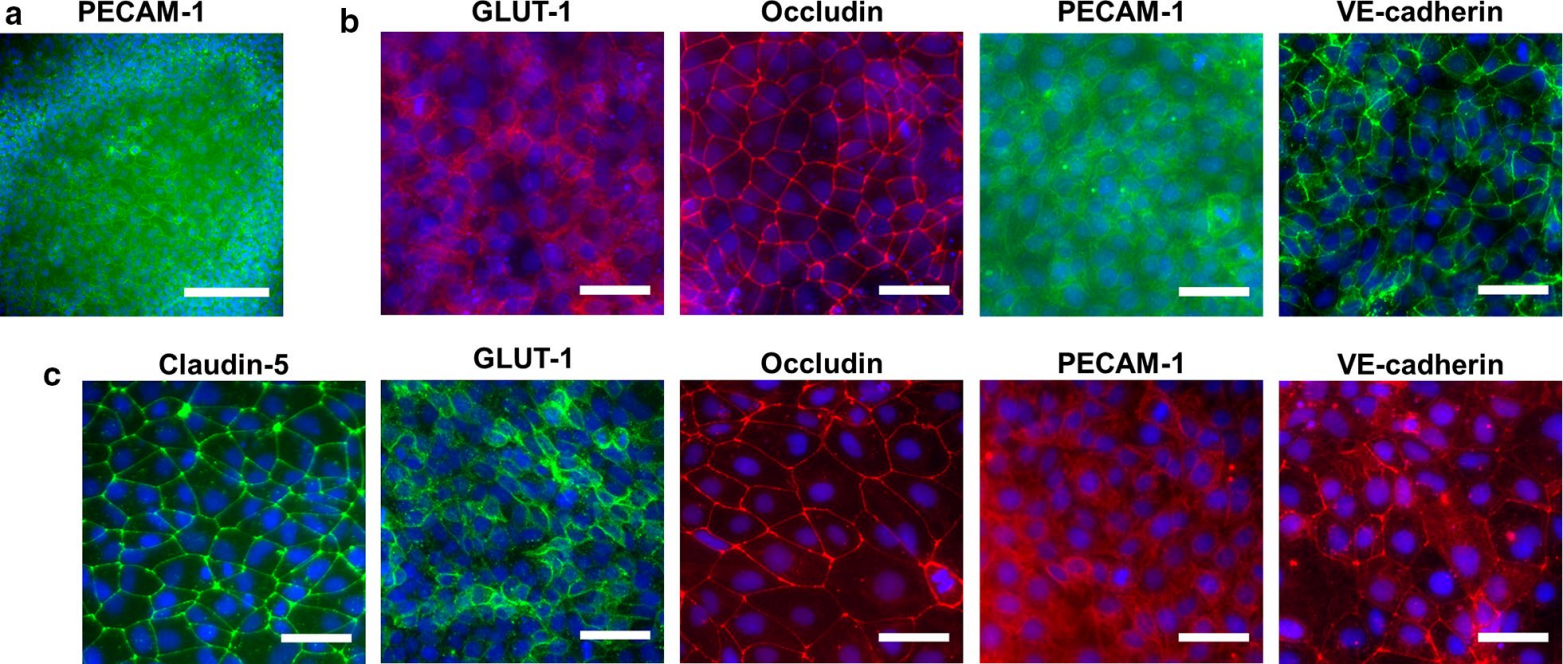
Immunocytochemical analysis of BBB markers before and after purification. **a** Immunocytochemical detection of PECAM-1 after 4 D E6 medium.* Scale bar* is 200 μm. **b** Immunocytochemical detection of GLUT-1, occludin, PECAM-1, and VE-cadherin after 4 D E6 medium and 2 D EC medium supplemented with bFGF and RA. All* scale bars* are 50 μm. **c** Immunocytochemical detection of claudin-5, GLUT-1, occludin, PECAM-1, and VE-cadherin following purification and induction of barrier phenotype. All* scale bars* are 50 μm

**Fig. 3:**
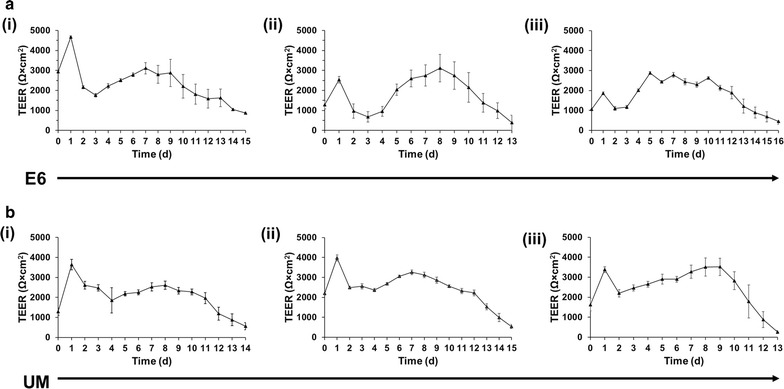
BMECs differentiated using UM and E6 medium demonstrate equivalent maximum TEER and stability. For each medium, three filters were seeded with BMECs, and TEER was measured in three different positions for each filter. Each plot is the result of one biological replicate (n = 1) with each daily TEER measurement the result of a technical n = 9. Values are reported as mean ± standard deviation of these collective measurements. **a** BMECs differentiated using E6 medium demonstrated maximum TEER values of at least 2500 Ω × cm^2^ for a minimum of 11 days in three biological replicates (*i*–*iii*). TEER was measured approximately every 24 h for the duration of the experiment. **b** BMECs differentiated using UM demonstrated maximum TEER values of at least 2500 Ω × cm^2^ for a minimum of 12 days in three biological replicates (*i*–*iii*). TEER was measured approximately every 24 h for the duration of the experiment

With E6 medium, we were surprised to observe large patches of PECAM-1^+^ endothelial cells after only 4 days of differentiation, which contrasts the time point when endothelium is first observed during UM differentiation (Fig. 2a) [24]. After 48 h in EC medium containing bFGF and RA, expression of PECAM-1, GLUT-1, VE-cadherin, and occludin were observed, indicating acquisition of a BBB phenotype (Fig. 2b). Cells were then purified onto Transwell filters or polystyrene culture plates using updated passaging techniques described by Wilson et al. [37]. Barrier phenotype was evaluated every 24 h by TEER, while immunocytochemistry was used to evaluate expression of endothelial and BBB markers 48 h after barrier induction. Immunocytochemical analysis of E6-derived BMECs showed robust expression of GLUT-1, claudin-5, occludin, VE-cadherin, and PECAM-1 (Fig. 2c), similar to UM-derived BMECs [24, 25]. E6-derived BMECs exhibited a maximum barrier of 4678 ± 49 Ω × cm^2^, and a minimum barrier stability of 1000 Ω × cm^2^ was observed over 12 days for three independent biological replicates (Fig. 3a). For comparison, UM-derived BMECs exhibited maximum TEER of 3980 ± 151 Ω × cm^2^ 24 h after barrier induction, a value comparable to previously published results [25], and maintained stability for a similar timeframe as E6-derived BMECs (Fig. 3b).

**Fig. 4:**
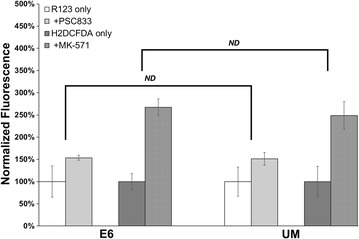
E6-derived BMECs and UM-derived BMECs demonstrate equivalent efflux transporter activity. E6-derived BMECs and UM-derived BMECs were incubated with rhodamine 123 (R123) or H_2_DCFDA in the presence and absence of efflux transporter inhibitors, PSC833 and MK-571, respectively. Fluorescence accumulation within cells incubated with fluorescent substrate in the presence of the desired inhibitor was normalized to fluorescence accumulation in cells incubated with substrate but with no inhibitor. Each condition was performed using triplicate wells, and cell count per condition was calculated as an average from 6 images taken from 1 additional well for each condition. All fluorescence values are normalized on per-cell basis and reported as normalized mean fluorescence ± standard deviation. Two-way ANOVA analysis indicates no statistical difference (p > 0.05) in fluorescence accumulation between E6-derived BMECs and UM-derived BMECs. All substrate and inhibitor conditions were repeated in an additional independent differentiation per medium to confirm reported trends

E6-derived BMECs were next evaluated for efflux transporter activity (ETA) and compared to UM-derived BMECs. Purified BMECs were incubated with rhodamine 123, a fluorescent substrate for P-glycoprotein, in the presence and absence of P-glycoprotein inhibitor PSC833. Fluorescence accumulation increased in the presence of PSC833 for both E6-derived BMECs (154 ± 5%) and UM-derived BMECs (151 ± 14%), which indicates inhibition of P-glycoprotein (Fig. 4). Purified BMECs were also incubated with H_2_DCFDA, a fluorescent MRP family substrate, in the presence and absence of MK-571, an MRP family inhibitor. Fluorescence accumulation increased in the presence of MK-571 for both E6-derived BMECs (267 ± 19%) and UM-derived BMECs (249 ± 31%), which indicates inhibition of MRP efflux transporters (Fig. 4). Differences in efflux transporter activity for the two sets of BMECs were statistically insignificant for both P-glycoprotein and the MRP family, suggesting that the change in differentiation medium did not have an effect on the efflux transporter activity of BMECs. Overall, these results indicate that using E6 medium to differentiate iPSCs to BMECs, in conjunction with updated seeding methods, reduces differentiation time and yields equivalent functionality as compared to established UM-based differentiation protocols.

### Defined medium supports BMEC differentiation in multiple iPSC lines

**Fig. 5:**
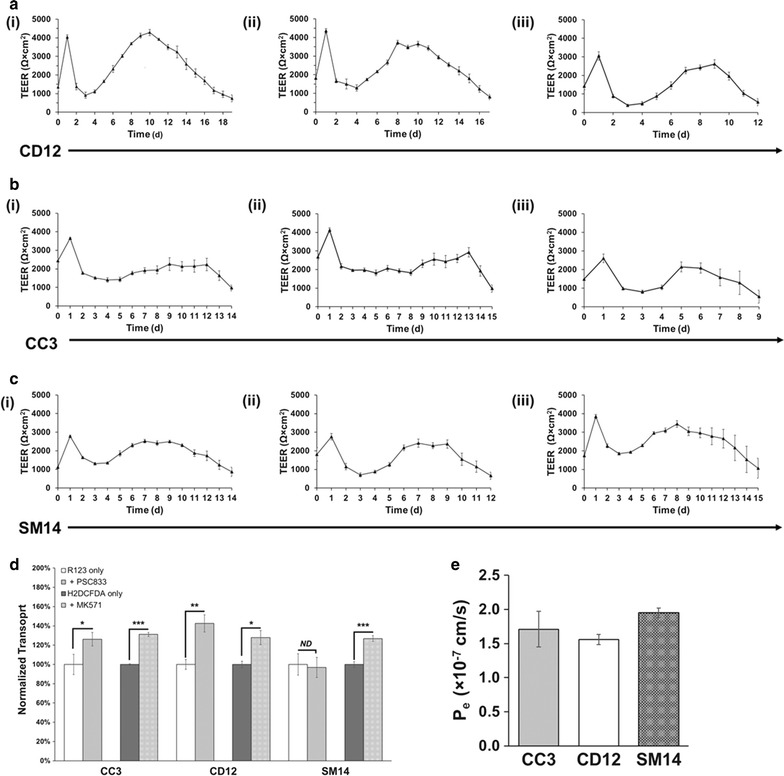
BMEC differentiation using E6 medium translates to additional iPSC lines. iPSC lines CD12, CC3, and SM14 were differentiated to BMECs with E6 medium as described in Fig. 1, and TEER was measured approximately every 24 h. For each differentiation, three filters were seeded with BMECs, and each filter was measured at three locations on the filter. Each plot is the result of one biological replicate (n = 1) with each daily TEER measurement the result of a technical n = 9. All values are mean ± standard deviation of these nine total measurements per condition. **a** CD12-derived BMECs achieved maximum TEER values exceeding 4000 Ω × cm^2^ and maintained TEER above 1000 Ω × cm^2^ for a minimum of 11 days in 3 independent biological replicates. **b** CC3-derived BMECs achieved maximum TEER values exceeding 3500 Ω × cm^2^ and maintained TEER above 1000 Ω × cm^2^ for a minimum of 8 days in 3 independent biological replicates. **c** SM14-derived BMECs achieved maximum TEER values exceeding 2500 Ω × cm^2^ and maintained TEER above 1000 Ω × cm^2^ for a minimum of 11 days in 3 independent biological replicates. **d** Apical-to-basolateral flux of rhodamine 123 (R123) and H_2_DCFDA was measured across BMECs in the presence or absence of PSC833 and MK-571, respectively. Fluorescence was normalized to cells not treated with inhibitor and reported as normalized mean fluorescence ± standard deviation. Each condition was performed using triplicate filters and statistics were calculated using a technical n of 3. Statistical significance was determined using the Student’s unpaired t test (*p < 0.1; **p < 0.05; ***p < 0.01). Data from one biological experiment are shown, and an additional biological replicate was performed for each line to verify the observed trends. **e** The permeability of sodium fluorescein was measured across BMECs. Each experiment was performed using triplicate filters and data are presented as mean ± standard deviation. Biological duplicates were used to verify each measurement. The effective permeability (P_e_) was calculated at less than 1.95 × 10^−7^ cm/s for all lines

After demonstrating the successful use of E6 medium to differentiate IMR90-4 iPSCs to BMECs, we sought to confirm the utility of this medium in other iPSC lines. Control iPSC lines CD12 and CC3 as well as iPSC line SM14, from a preclinical PD patient carrying compound heterozygous loss-of-function mutations in *PARK2* associated with familial early-onset PD [34–36], were differentiated to BMECs using E6 medium (Fig. 1). TEER measurements were used to assess barrier fidelity in three independent biological replicates for each line, and TEER exceeding 1000 Ω × cm^2^ was measured for a minimum of 8 days, with 5 out of 6 lines exhibiting stability above 1000 Ω × cm^2^ for at least 11 days (Fig. 5a-CD12, 5b-CC3, 5c-SM14). This barrier stability was comparable to results achieved in IMR90-4-derived BMECs differentiated in both E6 and UM. Notably, CD12-derived BMECs achieved TEER in excess of 4000 Ω × cm^2^ 10 days after purification, a maximum value that was also comparable to IMR90-4-derived BMECs.

Next, we sought to validate efflux transporter activity in these lines by conducting directional flux inhibition measurements. When treated with PSC833 and MK-571, CC3- and CD12-derived BMECs exhibited increased transport of corresponding substrate across the monolayer (Fig. 5d). SM14-derived BMECs, however, showed increased substrate transport across the barrier upon treatment with MK-571 but not PSC833, possibly indicating diminished P-glycoprotein function or polarization. Paracellular permeability of fluorescein (P_e_) was less than 1.95 × 10^−7^ cm/s for all lines, further confirming the passive barrier properties observed by TEER (Fig. 5e). These P_e_ measurements align with previously published values of BMECs derived using UM [30, 37].

### Co-culture with iPSC-derived astrocytes and primary human brain pericytes supports a sustained barrier phenotype

**Fig. 6:**
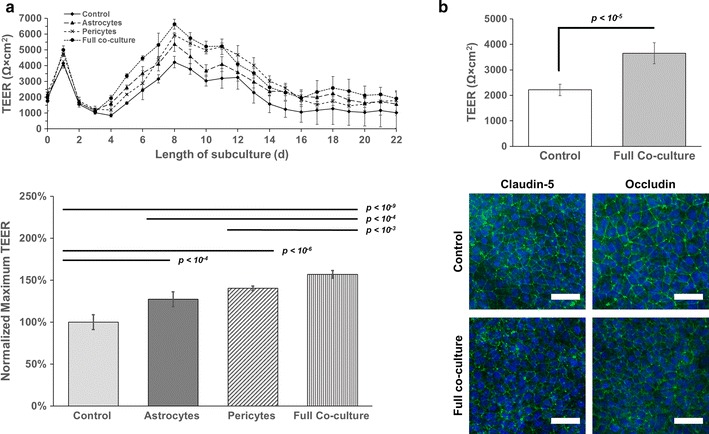
E6-derived BMECs respond to inductive cues from astrocytes and pericytes. **a** Co-culture of CD12-derived BMECs with astrocytes, pericytes, and a mixture of astrocytes and pericytes achieved maximum TEER values exceeding 4000 Ω × cm^2^ and maintained TEER above 1000 Ω × cm^2^ for a minimum of 22 days under all co-culture conditions. Each condition was conducted on triplicate filters with all BMECs purified from a single differentiation. Each filter was measured at three different locations on the filter each day. Values are mean ± standard deviation from these collective nine technical replicates per condition per day. Maximum TEER values achieved on day 8 of subculture were normalized to the TEER of the monoculture control. Statistical significance was calculated using Student’s unpaired t test. **b** CC3-derived BMECs were co-cultured with a mixture of astrocytes and pericytes, achieving a significant increase in TEER 24 h after barrier induction (p < 10^−5^, Student’s unpaired t test). Each condition was conducted on triplicate filters with all BMECs purified from a single differentiation. Each filter was measured at three different locations on the filter each day. Values are mean ± standard deviation from these collective nine technical replicates per condition per day. BMECs were subsequently stained for occludin and claudin-5 in both control and co-culture conditions.* Scale bars* are 50 μm

Induced pluripotent stem cell-derived BMECs have previously been shown to gain an elevated barrier phenotype when co-cultured with astrocytes and pericytes [25]. In order to evaluate if BMECs differentiated in E6 medium responded to such cues in a similar manner, iPSCs were differentiated to a mix of astrocytes and glial progenitors [28]. Co-culture of this cell population (49 ± 8% GFAP^+^, see Additional file 1: Figure S1) with CD12-derived BMECs was initiated 24 h after subculture onto Transwell filters, and co-culture was maintained in EC medium lacking bFGF and RA. Co-cultured cells were seeded in the bottom of the well plates; therefore, no direct contact occurred between BMECs and co-cultured cell types. As such, all observed differences are the result of soluble factors released in the system. Compared to BMECs in monoculture, BMECs in co-culture with glia reached both a higher absolute maximum TEER (5378 ± 479 Ω × cm^2^ versus 4227 ± 370 Ω × cm^2^, p < 10^−4^) and maintained TEER above 1000 Ω × cm^2^ in excess of 22 days (Fig. 6a). BMECs were also co-cultured with primary human brain pericytes (see Additional file 1: Figure S1) and achieved a maximum TEER of 5937 ± 157 Ω × cm^2^ (p < 10^−6^ versus monoculture) with a stable barrier phenotype above 1000 Ω × cm^2^ for longer than 22 days (Fig. 6a). BMECs co-cultured with both astrocytes and pericytes (35 ± 13% astrocytes, 63 ± 13% pericytes, Figure S1), achieved the highest maximum TEER of 6635 ± 315 Ω × cm^2^ (p < 10^−9^ versus monoculture, p < 10^−4^ versus astrocyte co-culture, p < 10^−3^ versus pericyte co-culture) and maintained TEER exceeding 1000 Ω × cm^2^ in excess of 22 days. These inductive effects were further verified through co-culture of CC3-derived BMECs with astrocytes and pericytes. A similar statistically significant increase in TEER was observed in co-cultured BMECs versus control BMECs, further validating the responsive nature of E6-derived BMECs to soluble cues from astrocytes and pericytes (Fig. 6b). Co-cultured and control BMECs grown on Transwell filters were stained for claudin-5 and occludin, and no significant phenotypic change was evident between monocultured BMECs on filters, BMECs in co-culture with astrocytes and pericytes, or BMECs grown in well plates (Fig. 2). Thus, the barrier-promoting effects of co-culture with astrocytes and pericytes exceeds effects of the two cell types alone, as expected from previous reports [25]. We note that media was not changed after day 0 to ascertain stability without any external stimulation, and we suspect that partial media exchanges could extend stability further. At present, we are also unsure why the TEER drops precipitously after day 1 and rebounds over the course of a week. We hypothesize this effect may be due to complete removal of bFGF, which is worthy of future exploration.

## Discussion

Protocols to differentiate iPSCs to BMECs for in vitro BBB modeling have not necessarily achieved widespread adoption, potentially due in part to the time required to produce purified BMECs and the cost associated with such differentiation and purification. The purpose of this study was to decrease both the time and cost required for such differentiation while still achieving BMECs of comparable performance to established differentiation methods. These advancements will potentially allow iPSC-derived BMECs to be more readily accessible to researchers, thereby providing high-fidelity human in vitro BBB models for a wide range of applications.

In this study, existing BMEC differentiation protocols were modified to exclude the iPSC expansion phase prior to initiation of differentiation [24, 25, 37]. By controlling initial iPSC seeding density [37], maximum TEER values of purified BMECs from such differentiations remained consistently in excess of 2500 Ω × cm^2^, and barrier fidelity, as indicated by TEER above 1000 Ω × cm^2^, was maintained for a minimum of 8 days, as was similarly achieved in a recent microfluidics-based model using UM-derived BMECs [40]. E8 medium was also used for iPSC maintenance in place of mTeSR, as described by others [30]. Given that we routinely use E6 medium (a derivative of E8 medium that lacks growth factors promoting pluripotency) for neural differentiations [28], we further explored its use for differentiating iPSCs to BMECs. Upon differentiation in E6 medium, immunocytochemical analysis unexpectedly showed PECAM-1^+^ cells at 4 days of differentiation rather than 6 days. We note that changes in culture methods and differentiation medium have previously been shown to alter differentiation times to lineages such as neuroectoderm [28, 41, 42] and midbrain dopaminergic neurons [41, 43], with all methods ultimately resulting in cells expressing the same characteristic markers. Therefore, it is unsurprising that changes made in differentiation medium resulted in altered differentiation timelines. After establishing this accelerated differentiation timeline from iPSCs to BMECs using E6 medium, BMECs were evaluated for BBB phenotype by TEER measurement and efflux transporter activity. BMECs differentiated in E6 medium maintained a stable barrier above 1000 Ω × cm^2^ for 8 days, longer than previously published reports using similar Transwell-based methods [25, 37], while achieving similar maximum TEER values to UM-derived BMECs. BMECs differentiated using E6 medium and UM also had no statistical difference in efflux transporter activity for P-glycoprotein and MRP family members. BMEC differentiation using E6 medium was further validated in iPSC lines CD12, CC3, and SM14. CD12- and CC3-derived BMECs demonstrated equivalent maximum TEER and long-term stability as IMR90-4-derived BMECs differentiated in both E6 medium and UM. Notably, CD12-derived BMECs achieved TEER greater than 4000 Ω × cm^2^ on days 9 and 10 of subculture, a value equivalent to the maximum TEER achieved after initial induction of BMEC phenotype. Finally, SM14 iPSCs, which harbor biallelic loss-of-function *PARK2* mutations associated with familial early onset PD, yielded BMECs with maximum TEER and long term stability equivalent to BMECs derived from control lines, similar fluorescein permeability, and equivalent MRP family efflux transporter activity. Interestingly, SM14-derived BMECs did not show active P-glycoprotein in the apical-to-basolateral transport assays. Studies on advanced PD patients have demonstrated increased brain uptake of P-glycoprotein substrates [44]. Our data may indicate that patients with familial PD mutations are predisposed to loss of P-glycoprotein function. However, our results are very preliminary and would need to be rigorously confirmed across multiple iPSC lines from different patients harboring the same mutation. As this manuscript is centered on the utility of E6 medium for in vitro differentiation, we have not pursued these studies herein. Even so, this exciting result indicates researchers can probe mechanisms of BBB regulation in the context of genetic disease or evaluate molecular transport and toxicology over extended experimental time points. Our methods can ostensibly be extended to other iPSC disease lines of interest, provided that the genetic mutation does not impact barrier stability.

To further explore the utility of the model, we investigated the effect of co-culturing BMECs with iPSC-derived astrocytes and primary human brain pericytes. Astrocytes and pericytes are known to aid in induction of BBB phenotype in the developing neurovascular environment [3]. Though co-culture of BMECs with astrocytes and co-culture of BMECs with pericytes individually were both found to increase barrier function as indicated by TEER, co-culture with astrocytes and pericytes concurrently enhanced the TEER above that achieved through co-culture with either cell type alone. This effect has been noted in other in vitro BBB models, though the reported maximum TEER value from co-culture in this study exceeds previously published in vitro TEER values by more than 700 Ω × cm^2^ [25]. Excitingly, this achievement approaches in vivo TEER predictions of 8000 Ω × cm^2^ put forth by Smith and Rapoport [45]. In addition, medium was not changed following barrier induction to minimize external influences on barrier stability. Due to prospective increased metabolic burden in the co-culture system as evidenced by a qualitative decrease in number of co-cultured cells between the start and end of the experiment, we suspect that gradual media changes following barrier induction may further improve the stability of the model by supporting neurovascular health. Owing to the extended barrier phenotype observed, researchers may now conduct long-term experiments without concern that barrier degradation may confound results.

## Conclusions

The differentiation of iPSCs to BMECs using defined E6 medium shortened differentiation time and produced BMECs of equivalent performance as BMECs differentiated according to previously published protocols using UM. Stability of the model system has also been greatly improved, with monocultured BMECs exhibiting enhanced stability for greater than 8 days and co-cultured BMECs exhibiting stability of more than 3 weeks. We routinely produce E6 and E8 medium in-house in large batches (see “Methods”), thus substantially reducing the cost of maintenance and differentiation to allow increased throughput of experiments. Thus, the overall improvements described in this manuscript will enable iPSC-derived BMECs to become more accessible to researchers and more broadly enable studies ranging from disease pathogenesis to analyses of repeated drug dosing.

## Additional file

**Additional file 1.** Additional characterization of iPSC-derived BMECs–primary antibody and secondary antibody information, including recognized antigen, clone, vendor, and dilution, are listed in Tables S1 and S2, respectively. Immunocytochemical analysis of astrocytes and pericytes used in co-culture experiment are shown in Figure S1.

## Abbreviations

BBB: blood–brain barrier
BMEC: brain microvascular endothelial cell
iPSC: induced pluripotent stem cell
H_2_DCFDA: 2′,7′-dichlorodihydrofluorescein diacetate
R123: rhodamine 123
EC: endothelial cell
UM: unconditioned medium
MRP: multidrug resistance protein
RA: retinoic acid
bFGF: basic fibroblast growth factor
TEER: trans-endothelial electrical resistance
ETA: efflux transporter activity
ICC: immunocytochemistry
CNTF: ciliary neurotrophic factor
EGF: epidermal growth factor
TGFβ1: transforming growth factor beta-1
PD: Parkinson’s Disease
