# Supplementary material for: Accelerated differentiation of human induced pluripotent stem cells to blood–brain barrier endothelial cells
Source: Fluids Barriers CNS. 2017 Apr 13;14:9. doi: 10.1186/s12987-017-0059-0 (PMC5390351; doi:10.1186/s12987-017-0059-0)
Supplement: Supplementary file 1 — Additional file 1. Additional characterization of iPSC-derived BMECs–primary antibody and secondary antibody information, including recognized antigen, clone, vendor, and dilution, are listed in Tables S1 and S2, respectively. Immunocytochemical analysis of astrocytes and pericytes used in co-culture experiment are shown in Figure S1. [file 12987_2017_59_MOESM1_ESM.docx]

**Supplementary Information**

**Accelerated differentiation of human induced pluripotent stem cells to blood-brain barrier endothelial cells**

Emma K. Hollmann, Amanda K. Bailey, Archit V. Potharazu, M. Diana Neely, Aaron B. Bowman, and Ethan S. Lippmann

**Inventory of supplementary information**

Table S1

Table S2

Figure S1

**Table S1: Primary antibodies used in immunocytochemistry experiments**

| **Target Antigen** | **Antibody Species** | **Vendor** | **Clone or product number** | **Dilution** |
| --- | --- | --- | --- | --- |
| PECAM-1 | Rabbit | Thermo Fisher Scientific | RB-10333-P | 1:25 |
| Claudin-5 | Mouse | Thermo Fisher Scientific | 4C3C2 | 1:50 |
| Occludin | Mouse | Thermo Fisher Scientific | OC-3F10 | 1:100 |
| VE-Cadherin | Goat | R&D Systems | AF938 | 1:100 |
| GLUT-1 | Mouse | Thermo Fisher Scientific | SPM498 | 1:50 |
| Glial fibrillary acidic protein (GFAP) | Rabbit | Dako | Z0334 | 1:500 |
| Platelet-derived growth factor β (PDGFRβ) | Rabbit | Santa Cruz Biotechnology | sc-432 | 1:100 |
| NG2 | Mouse | Santa Cruz Biotechnology | sc-53389 | 1:50 |
| SMA | Mouse | Santa Cruz Biotechnology | sc-130616 | 1:100 |

**Table S2: Secondary antibodies used in immunocytochemistry experiments**

| **Species Reactivity** | **Host** | **Conjugate** | **Vendor** | **Dilution** |
| --- | --- | --- | --- | --- |
| Rabbit | Donkey | Alexa Fluor 488 | Thermo Fisher Scientific | 1:200 |
| Rabbit | Donkey | Texas Red | Thermo Fisher Scientific | 1:200 |
| Mouse | Donkey | Alexa Fluor 488 | Thermo Fisher Scientific | 1:200 |
| Mouse | Donkey | Texas Red | Thermo Fisher Scientific | 1:200 |
| Goat | Donkey | Alexa Fluor 488 | Thermo Fisher Scientific | 1:200 |
| Goat | Donkey | Texas Red | Thermo Fisher Scientific | 1:200 |

**
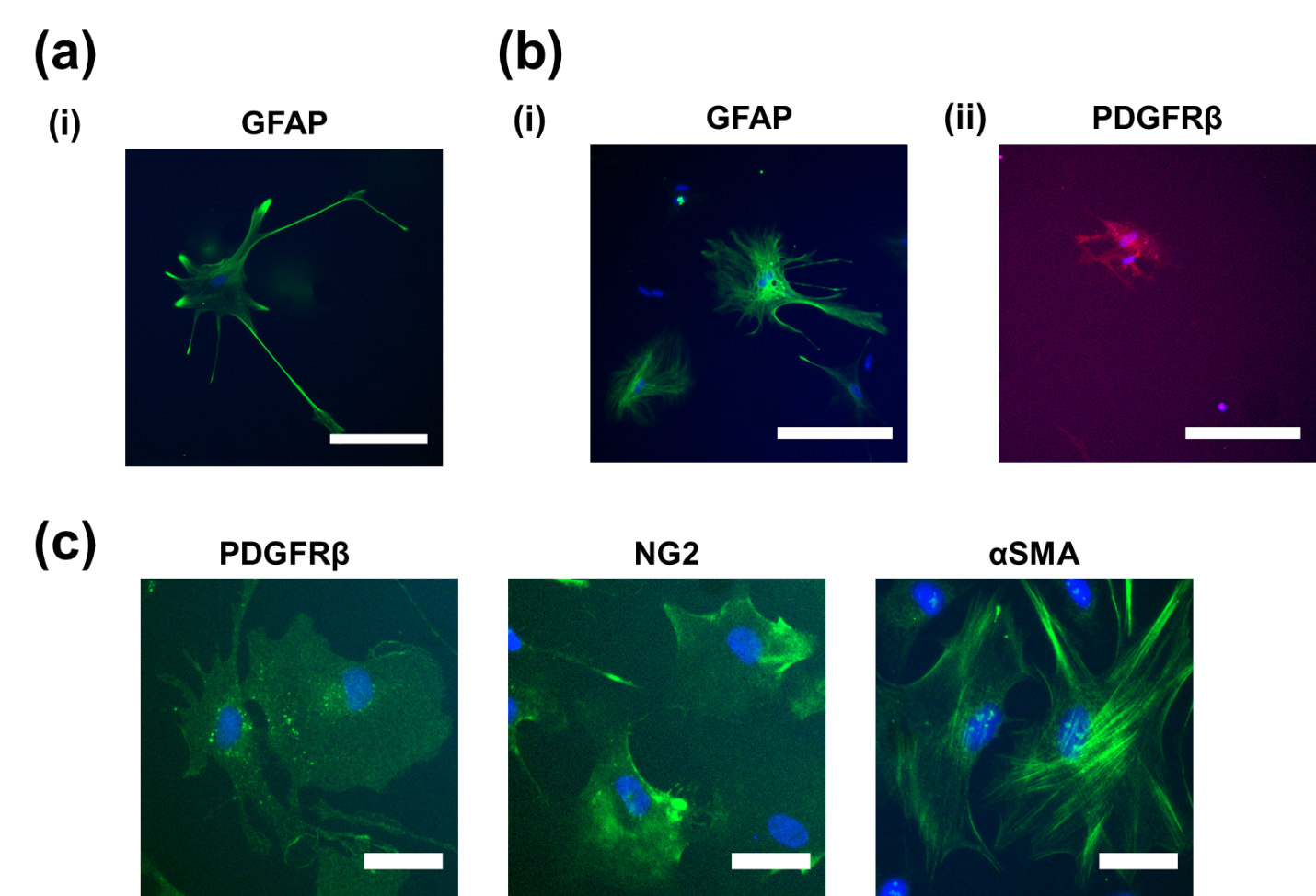
**

**Figure S1: Characterization of astrocytes and pericytes in coculture with iPSC-derived BMECs.** (a) IMR90-4 iPSC-derived astrocytes were fixed upon termination of the coculture experiment, and the impure populations were stained for (i) GFAP. (b) IMR90-4 iPSC-derived impure astrocytes and primary brain pericytes were fixed upon termination of the coculture experiment. Astrocytes were stained for (i) GFAP, and pericytes were stained for (ii) PDGFRβ. Percentage of GFAP^+^ and PDGFRβ^+^ cells in (a) and (b) was determined by normalizing GFAP^+^ and PDGFRβ^+^ positive cells to total nuclei count in 6 frames per stain imaged, and scale bars 200 μm. (c) Primary brain pericytes, absent co-culture with BMECs, were stained for PDGFRβ, NG2, and αSMA at passage 15 to verify identity. Scale bars are 50 μm.
